# α-Hederin inhibits the growth of lung cancer A549 cells *in vitro* and *in vivo* by decreasing SIRT6 dependent glycolysis

**DOI:** 10.1080/13880209.2020.1862250

**Published:** 2020-12-27

**Authors:** Cong Fang, Yahui Liu, Lanying Chen, Yingying Luo, Yaru Cui, Ni Zhang, Peng Liu, Mengjing Zhou, Yongyan Xie

**Affiliations:** a National Pharmaceutical Engineering Center for Solid Preparation in Chinese Herbal Medicine, Jiangxi University of Traditional Chinese Medicine, Nanchang, China; b College of Traditional Chinese Medicine, Jiangxi University of Traditional Chinese Medicine, Nanchang, China

**Keywords:** Antitumor, Warburg effect, glucose, c-Myc, HIF-1α

## Abstract

**Context:**

α-Hederin, a potent bioactive compound of *Pulsatilla chinensis* (Bunge) Regel (Ranunculaceae), has many pharmacological uses, but its effect on cancer cell metabolism is still unclear.

**Objective:**

To elucidate the role of α-hederin in the glucose metabolism of lung cancer cells.

**Materials and methods:**

Cell Counting Kit 8 and colony formation assays were employed to assess the antiproliferative effects of α-hederin. Glucose uptake, ATP generation, and lactate production were measured. Glycolysis-related proteins were detected using western blotting, and a sirtuin 6 (SIRT6) inhibitor was used to verify A549 cell proliferation. Sixty male BALB/c nude mice were divided into normal control, 5-FU (25 mg/kg), and α-hederin (5 and 10 mg/kg) groups to assess the antitumor effect for 32 days. Glycolysis-related protein expression was evaluated using immunohistochemical analysis.

**Results:**

α-Hederin inhibited A549 (IC_50_ = 13.75 μM), NCI-H460 (IC_50_ = 17.57 μM), and NCI-H292 (IC_50_ = 18.04 μM) proliferation; inhibited glucose uptake and ATP generation; and reduced lactate production. Furthermore, α-hederin (10 and 15 μM) markedly inhibited hexokinase 2, glucose transporter 1, pyruvate kinase M2, lactate dehydrogenase A, monocarboxylate transporter, c-Myc, hypoxia-inducible factor-1α, and activated SIRT6 protein expression. Using a SIRT6 inhibitor, we demonstrated that α-hederin inhibits glycolysis by activating SIRT6. A tumour xenograft mouse model of lung cancer confirmed that α-hederin (5 and 10 mg/kg) inhibits lung cancer growth by inhibiting glycolysis *in vivo.*

**Discussion and conclusions:**

α-Hederin inhibits A549 cell growth by inhibiting SIRT6-dependent glycolysis. α-Hederin might serve as a potential agent to suppress cancer.

## Introduction

In recent years, the incidence of lung cancer has been increasing. Lung cancer is the deadliest form of cancer and is responsible for about a quarter of all cancer deaths (Siegel et al. 2020). Lung cancer includes primarily two types: small cell lung cancer and non-small cell lung cancer (NSCLC), the latter of which accounts for 85% of all lung cancer cases. Because early symptoms are not obvious, approximately 70% of patients with NSCLC are diagnosed with advanced lung cancer and the 5-year survival rate is only 19% (Siegel et al. 2020). Early lung cancer patients can choose to receive chemotherapy and surgery at the same time, which is the current recommended standard treatment, but chemotherapy often causes many adverse reactions. However, improvement in treatment modalities, including surgery, chemotherapy, and radiation therapy, has progressed (Molina et al. 2008; Gomez-Casal et al. 2013; Yuan et al. 2014). Nevertheless, despite these advances, the prognosis for NSCLC has not improved. Therefore, additional therapeutic strategies are needed.

**Figure 1.**
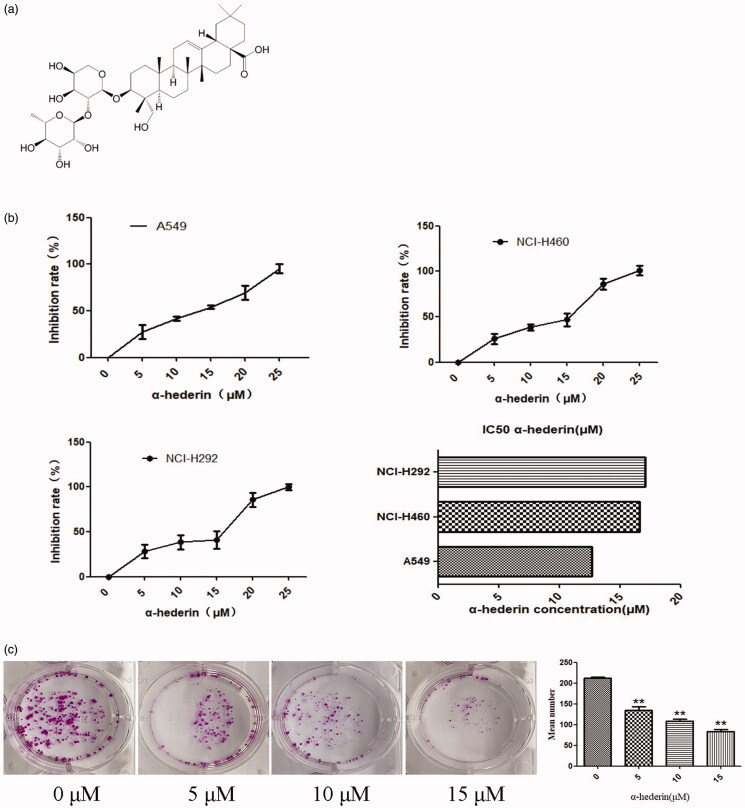
α-Hederin inhibits proliferation of human lung cancer cells. (a) Chemical structure of α-Hederin. (b) Effect of α-hederin on the viability of human lung cancer cells. A549, NCI-H460, and NCI-H292 cells were treated with α-hederin for 48 h. CCK8 assay was performed to analyse cell viability. Results are normalized to PBS controls. (c) Effect of α-Hederin on the viability of non-small cell lung cancer A549 cells. Colony formation of A549 cells treated with α-hederin for 48 h, cultured by DMEM for 10 days, and stained with crystal violet. **p < 0.01 as compared to the 0 μM group. Mean ± S.E.M. *n* = 3.

*Pulsatilla chinensis* (Bunge) Regel (Ranunculaceae), which is used for its cooling and detoxifying abilities, has been reported to have many pharmacological characteristics such as being antimalarial and antibacterial (Cheng et al. 2008) and an anticancer agent (Liu et al. 2013). In our previous study, we demonstrated that three monomers of *Pulsatilla chinensis*, PSA, R13, and PSD, can considerably inhibit the proliferation of tumour cell lines (Guan et al. 2020). α-Hederin (Figure 1(a)) is a monosaccharide chain pentacyclic triterpenoid saponin and is one of the effective components of the traditional Chinese medicine *Pulsatilla chinensis*. Studies have found that α-hederin plays many pharmacological roles, including antitumor, antifungal, insecticidal, antispasmodic, analgesic, and hepatic protection (Shi and Liu 1996; Ridoux et al. 2001; Li et al. 2003; Rooney and Ryan 2005; Sieben et al. 2009).

Reprogramming energy metabolism is a hallmark of cancer. Energy metabolism is the process in which energy is generated from nutrients, released, stored, and consumed by organisms or living cells. Energy metabolism is divided into glucose metabolism, protein metabolism, and fat metabolism. Under normal conditions, cells generate energy primarily via aerobic respiration. When the oxygen content is insufficient, cells perform glycolysis to generate energy. This process is called anaerobic respiration. Unlike normal cells, tumour cells generate energy primarily via glycolysis, even under aerobic conditions, a phenomenon known as the Warburg effect. Glycolytic capacity is characterized by rapid productivity and low efficiency. The rapid proliferation of tumour cells requires rapid energy consumption. Meanwhile, the lactic acid generated by glycolysis creates an acidic environment for tumour cells, which is conducive to their growth and leads to their rapid proliferation (Zhao et al. 2014; Potter et al. 2016). Sirtuin 6 (SIRT6) protein is a chromatin binding factor that was initially described as an inhibitor of gene instability (Mostoslavsky et al. 2006). During energy metabolism, SIRT6 regulates the fat and glucose metabolism, which is a key regulator of energy stress and is closely related to the process of tumour growth (Sebastián and Mostoslavsky 2015). With the metabolic profile used for energy production is elucidated, regulating tumour metabolism is a new therapeutic strategy to inhibit tumour growth (Zhang and Yang 2013).

We used *in vitro* and *in vivo* studies of α-hederin to address NSCLC inhibition and determine glucose in glycolytic tumour metabolism, lactate and ATP content, key enzymes in the process of glycolytic metabolism, and relevant transporters. We also assessed the regulatory factors involved in glycolysis to clarify the effect of α-hederin on the inhibition of NSCLC.

## Materials and methods

### Chemicals and reagents

α-Hederin (purity 99%) was provided by Chengdu Push Bio-Technology Co. Ltd. (Chengdu, China) and the cell count kit 8 (CCK8) was purchased from Dojindo Laboratories (Kumamoto, Japan). RPMI-1640 and Dulbecco’s modified Eagle’s medium (DMEM) were purchased from HyClone Laboratories (Logan, UT, USA). Foetal bovine serum (FBS) was obtained from Biological Industries (Beit HaEmek, Israel). 2-Deoxy-d-glucose (2DG) and SIRT6 inhibitor OSS_128167 were purchased from MedChemExpress (Monmouth Junction, NJ, USA). Hexokinase 2 (HK2), glucose transporter 1 (GLUT1), pyruvate kinase M2 (PKM2), monocarboxylate transporter (MCT4), lactate dehydrogenase A (LDHA), c-Myc, p53, hypoxia-inducible factor-1α (HIF-1α), protein kinase B (Akt), and p-Akt antibodies were purchased from Abcam (Cambridge, UK). SIRT6 antibody was purchased from Proteintech (Rosemont, IL, USA).

### Cell culture

Human NSCLC A549 cells and human lung cancer cells NCI-H460 and NCI-H292 (lymph node metastasis) were provided by the Stem Cell Bank of the Chinese Academy of Sciences. NCI-H460 and NCI-H292 cells were cultured in RPMI-1640 medium containing 10% FBS, and A549 cells were cultured in DMEM containing 10% FBS in a 5% CO_2_ incubator at 37 °C.

### Cell viability assay

The effect of α-hederin on the proliferation of A549, NCI-H292, and NCI-H460 cells was assessed using the CCK8 assay. Cell suspensions were seeded into 96-well plates at 5000 cells/well. After incubation for 24 h, the cells were treated with α-hederin (0, 5, 10, 15, 20, and 25 μM) for 48 h. Subsequently, 10 μL of CCK8 was added to each well and the plate was incubated for 1 h. Absorbance was measured at 450 nm using a SpectraMax® i3 Multi-mode Microplate Reader (Molecular Devices, San Jose, CA, USA). The inhibition rate was calculated using the following formula:

$$\text{Inhibition Rate }(\%)=(1-{\mathrm{Absorbance}}_{\text{treated group}}/{\mathrm{Absorbance}}_{\text{control group}})\times 100\%.$$

### Colony formation assay

The effect of α-hederin on cell proliferation was detected via colony formation assay. A549 cells were seeded into 6-well plates (500 cells/well) and incubated for 24 h, after which the cells were treated with 5, 10, or 15 μM α-hederin for 48 h. The cells were then cultured in DMEM containing 10% FBS for 10 days, followed by fixation with 4% formaldehyde and staining with 0.5% crystal violet. The number of colonies was quantified.

### Glucose uptake, lactate, and ATP assays

A549 cells were cultured in 6-well plates (2 × 10^5^ cells/well) and incubated for 24 h. Then DMEM containing 10% FBS and 0, 5, 10, or 15 μM α-hederin were seeded into the 6-well plates, the plates were incubated for 24 h, and culture media were collected. Glucose, lactate, and ATP levels were assayed using a kit (Jiancheng, Nanjing, China) following the manufacturer’s instructions. Absorbance was determined using a microplate reader, and the amount of glucose uptake, lactate generated, and ATP levels in the cells per protein mass were determined.

### Western blotting

For western blot analysis, A549 cells were placed in 6-cm dishes (1 × 10^6^ cells/dish), some of which were treated with α-hederin for 24 h. The cells were then lysed in RIPA buffer containing protease and phosphatase inhibitors (Thermo Fisher Scientific, Waltham, MA, USA). Proteins were separated out using SDS-PAGE based on molecular weight and blotted onto a polyvinylidene fluoride membrane (Thermo Fisher Scientific). Bands were incubated with the primary antibodies HK2 (1:2000), GLUT1 (1:10,000), PKM2 (1:1000), MCT4 (1:1000), LDHA (1:1000), c-Myc (1:1000), p53 (1:1000), HIF-1α (1:1000), Akt (1:1000), p-Akt (1:1000), and SIRT6 (1:1000) overnight at 4 °C and measured using a ChemiDoc Imaging System with Image Lab Software version 4.1 (Bio-Rad, Hercules, CA, USA).

### Animal models

Six-week-old male BALB/c nude mice (Hunan SJA Laboratory Animal Co. Ltd., China) were used as an A549 xenograft tumour mouse model. The mice were housed in a pathogen-free environment at the institutional animal care facility at 21 °C and relative humidity of 55% with free access to food and water. The mice were injected with 5 × 10^6^ A549 cells subcutaneously. After the tumour reached a certain size, it was removed and stripped clean, and tumour tissue (approximately 3 × 3 × 3 mm^3^) was detached using scissors. A small incision was made on the lateral abdomen of the recipient animals and a small piece of tumour tissue was implanted into the right armpit using unhooked ophthalmic tweezers. The blank group of mice was injected with culture medium when the tumour volume reached 100 mm^3^. Then the mice were randomly assigned to five groups and treated intraperitoneally with α-hederin (10 or 5 mg/kg), 5-FU (25 mg/kg), or a vehicle every other day. The mice were euthanized after 32 days of treatment and tumour volume (length × width^2^ × 0.5236) and body weight were determined. Finally, the tumours were fixed in 4% paraformaldehyde.

The procedures for the care and use of the animals were approved by the experimental animal ethics committee of Jiangxi University of Traditional Chinese Medicine (JZLLSC2018-0053), and all applicable institutional and governmental regulations concerning the ethical use of animals were followed.

### Immunohistochemical analysis

Tumour tissues were embedded in paraffin and cut into 5-μm sections using a microtome (Leica Biosystems, Wetzlar, Germany). Following the instructions of the manufacturer (Cowin Biosciences, Taizhou, Jiangsu, China), tumour sections were incubated with GLUT1 (1:400), HK2 (1:400), PKM2 (1:30), LDHA (1:400), MCT4 (1:100), HIF-1α (1:200), c-Myc (1:100), and SIRT6 (1:100) antibodies overnight at 4 °C. Tumours were taken from each group, 3 sections were taken from each tumour, and 6 fields were chosen from each slice. The results were calculated using the mean integrated optical density and analysed using Image-Pro Plus 6.0 (Media Cybernetics, Rockville, MD, USA).

### Statistical analyses

Significant differences between individual groups were determined by comparison using one-way ANOVA. A *t*-test was used for statistical analyses between two independent groups. All tests were performed using IBM SPSS Statistics 19 (IBM Corp., Armonk, NY, USA), and *p* < 0.05 was considered significant.

## Results

### α-Hederin inhibits proliferation of human lung cancer cells

Our CCK8 results showed that α-hederin had significant inhibitory effects on the proliferation of human lung cancer cells. The IC_50_ of α-hederin in NSCLC A549, NCI-H460, and NCI-H292 was 13.75, 17.57, and 18.04 μM, respectively. α-Hederin exhibited a superior inhibitory effect on A549 cells, which subsequently were used for experiments (Figure 1(b)).

The colony formation assay demonstrated that α-hederin inhibited the clonogenic effects of A549 cells. Compared to 0 µM α-hederin, 5, 10, and 15 μM α-hederin significantly decreased the number of cells (*p* < 0.05), with 15 μM α-hederin having the best inhibitory effect (Figure 1(c)).

### α-Hederin inhibits glycolytic metabolism in human NSCLC A549 cells

**Figure 2.**
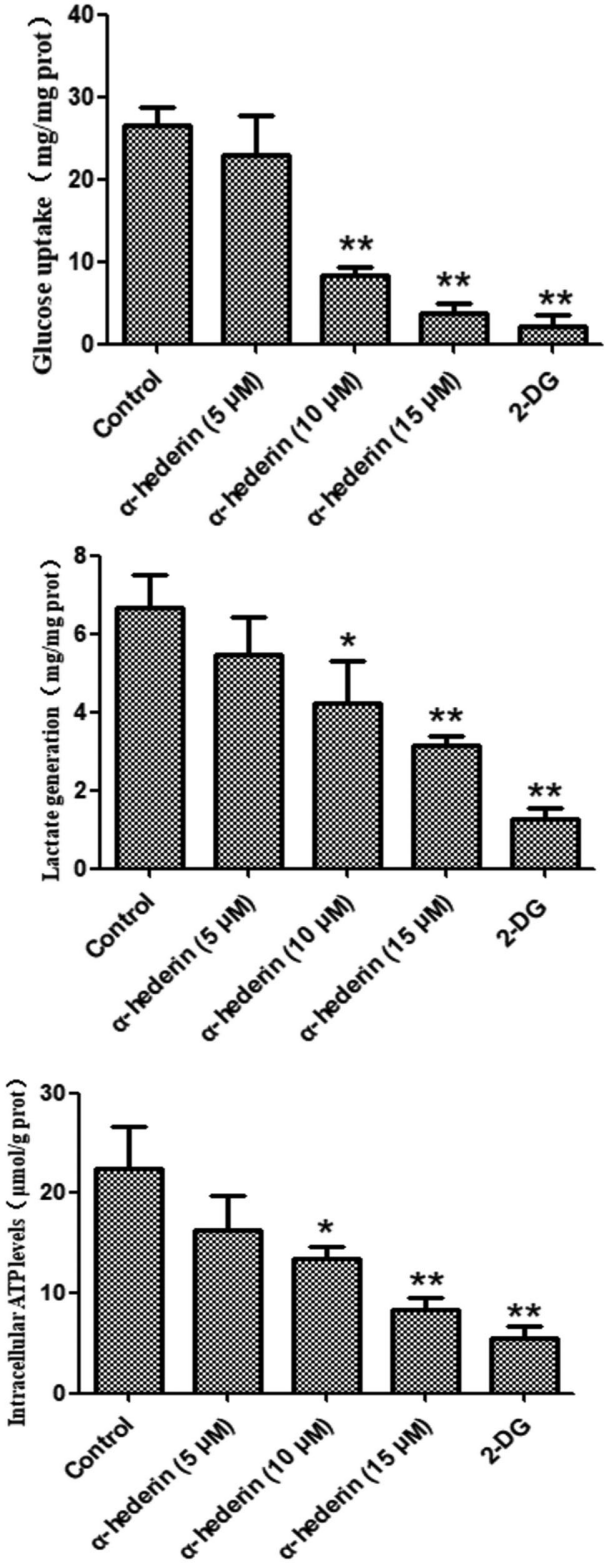
α-Hederin dramatically inhibits aerobic glycolysis in A549 cancer cells. Glucose uptake, lactate generation, and intracellular ATP levels in A549 cells in response to α-hederin treatment for 48 h. The concentration of 2-DG was 8 mM. *p < 0.05, **p < 0.01 as compared to the control group. Mean ± S.E.M. *n* = 3.

Glycolytic metabolic levels can be assessed by glucose uptake, lactate production, and ATP levels. 2DG, an inhibitor of glucose metabolism, was used as a positive control. Results revealed that compared to glucose consumption by the control group (0 μM α-hederin), glucose consumption by A549 cells treated with 2DG and 10 and 15 μM α-hederin was significantly lower (*p* < 0.01) (Figure 2). Compared to lactose production in the control group (0 μM α-hederin), lactate production in A549 cells treated with 2DG and 10 and 15 μM α-hederin was significantly reduced (*p* < 0.05) (Figure 2). Compared to ATP levels in the control group (0 μM α-hederin), the ATP levels in A549 cells treated with 2DG and 10 and 15 μM α-hederin were significantly reduced (*p* < 0.05) (Figure 2).

### α-Hederin inhibits glycolysis-related proteins in human NSCLC A549 cells

**Figure 3.**
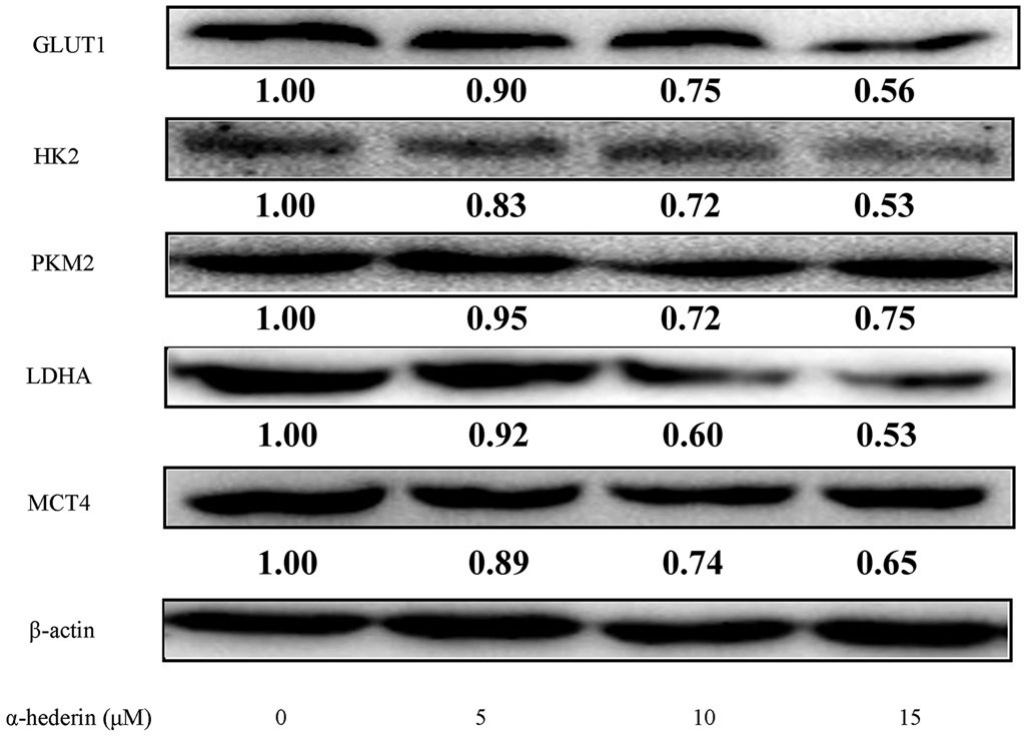
α-Hederin inhibits glycolytic related proteins in human non-small cell lung cancer A549 cells. A549 cells were treated with α-hederin for 24 h. Expression of GLUT1, HK2, PKM2, LDHA and MCT4 was detected by Western blot.

GLUT1 transports extracellular glucose into the cell, which then generates lactate and ATP via the catalysis of enzymes, including HK2, PKM2, and LDHA. Lactate and ATP are used in tumour cell proliferation. Finally, MCT4 transports intracellular lactate out of the cell. Western blot results showed that compared to 0 µM α-hederin, 10 and 15 μM α-hederin significantly reduced the expression of GLUT1, HK2, PKM2, LDHA, and MCT4 (Figure 3).

### α-Hederin inhibits glycolysis-related proteins in A549 cells by inhibiting c-Myc and HIF-1α expression

**Figure 4.**
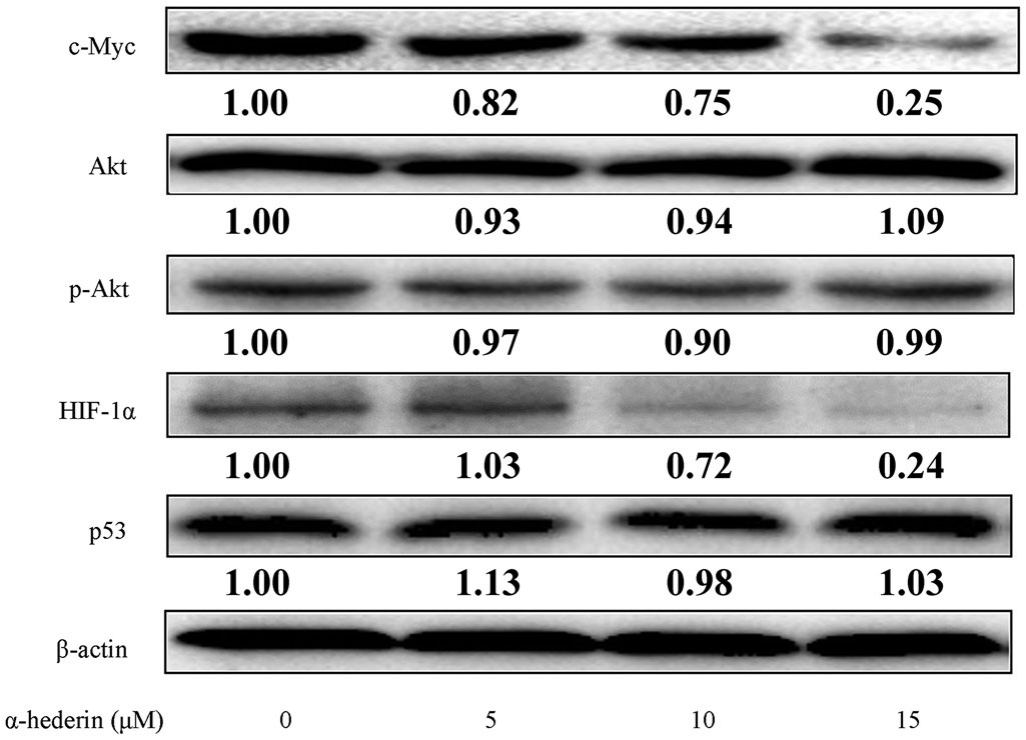
α-Hederin reduces glycolytic levels by inhibiting c-Myc and HIF-1α in human non-small cell lung cancer A549 cells. A549 cells were treated with α-hederin for 24 h. Expression of c-Myc, Akt, HIF-1α, and p53 was detected by western blots.

c-Myc, HIF-1α, Akt, and p53 have distinct effects on proteins involved in glycolysis in tumour cells. The results of our study showed that each α-hederin dose had no significant effect on the expression of glycolysis regulators Akt and p53 protein in tumour cells, while the expression of c-Myc and HIF-1α was significantly downregulated in each α-hederin group compared to the 0 μM α-hederin group (Figure 4). Expression of HIF-1α was significantly downregulated with 10 and 15 μM α-hederin, suggesting that α-hederin may inhibit aerobic glycolysis in tumour cells by regulating the expression of c-Myc and HIF-1α (Figure 4).

### α-Hederin inhibits c-Myc and HIF-1α by activating SIRT6 expression

**Figure 5.**
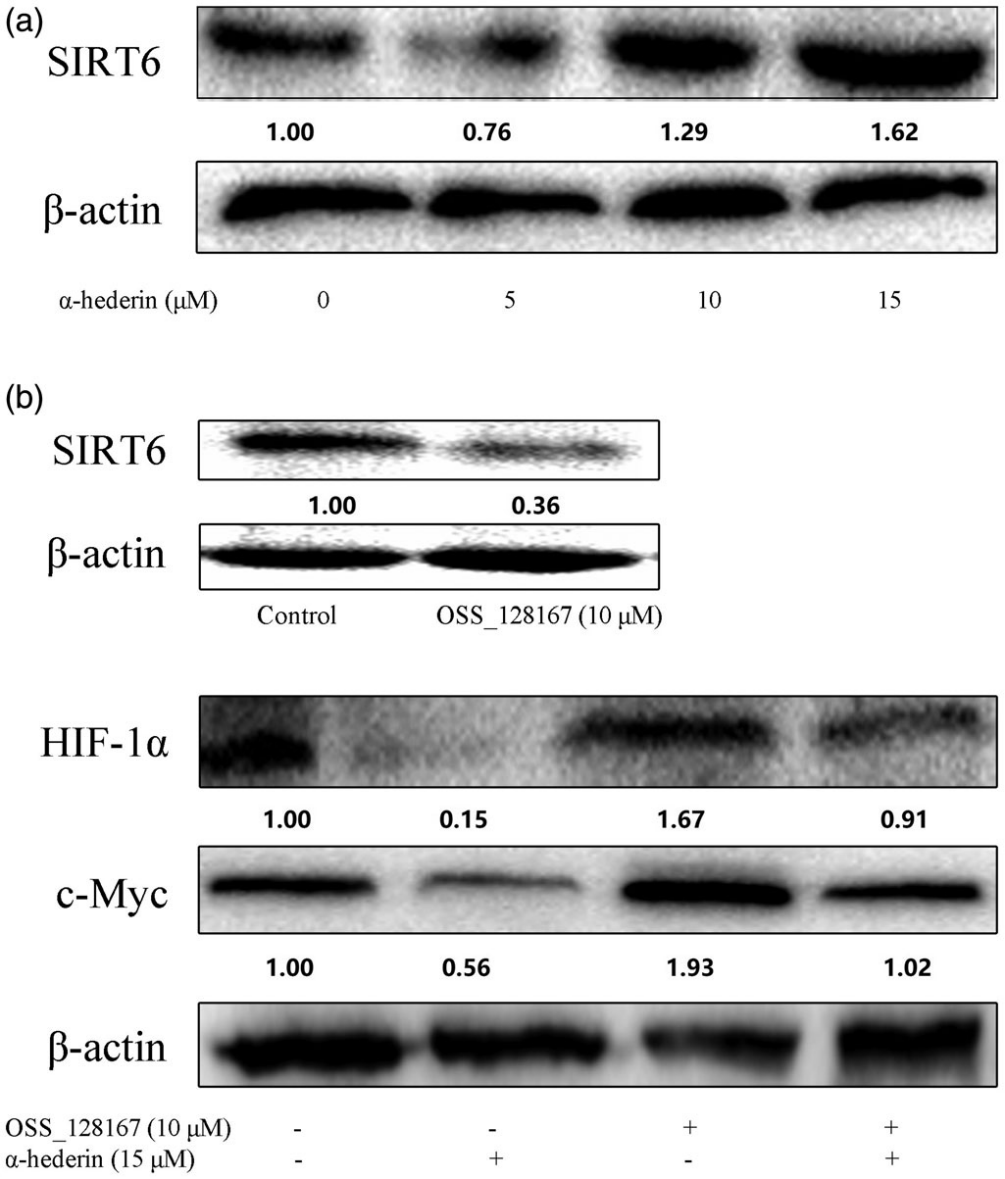
α-Hederin inhibits c-Myc and HIF -1α by activating expression of SIRT6. (a) A549 cells were treated with α-hederin for 24 h. Expression of SIRT6 was detected by western blot. (b) A549 cells were treated with α-hederin or SIRT6 inhibitor OSS_128167 for 24 h. Expression of c-Myc and HIF-1α was detected by western blots.

Our results showed that SIRT6 expression was significantly upregulated in each group compared to that in the control (Figure 5(a)). After the addition of the SIRT6 inhibitor OSS_128167, SIRT6 protein levels were significantly downregulated and the expression of glycolysis-related proteins c-Myc and HIF-1α was upregulated compared to that in the control. When α-hederin and OSS_128167 were combined, the expression of c-Myc and HIF-1α was upregulated compared to their expressions when OSS_128167 and α-hederin were used alone (Figure 5(b)).

### Effect of α-hederin combined with OSS_128167 on A549 cell viability

**Figure 6.**
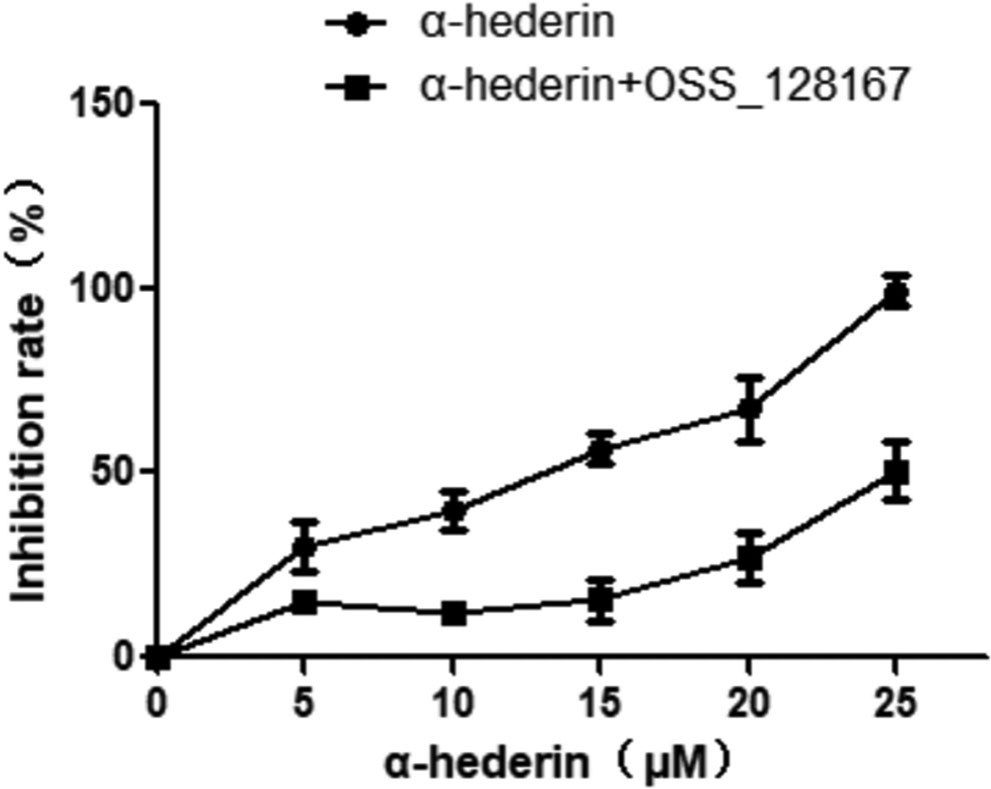
Effect of α-hederin combined with SIRT6 inhibitor OSS_128167 on the viability of A549 cells. Cells were treated with α-hederin for 48 h. CCK8 assay was performed to analyse cell viability. Results are normalized to PBS controls.

We examined the inhibitory effect of α-hederin combined with the SIRT6 inhibitor OSS_128167 on the proliferation of A549 cells. CCK8 results showed that compared to the inhibitory effect of α-hederin alone, that of α-hederin combined with OSS_128167 was significantly decreased (Figure 6).

### Inhibitory effect of α-hederin on xenograft NSCLC A549 tumours in nude mice

**Figure 7.**
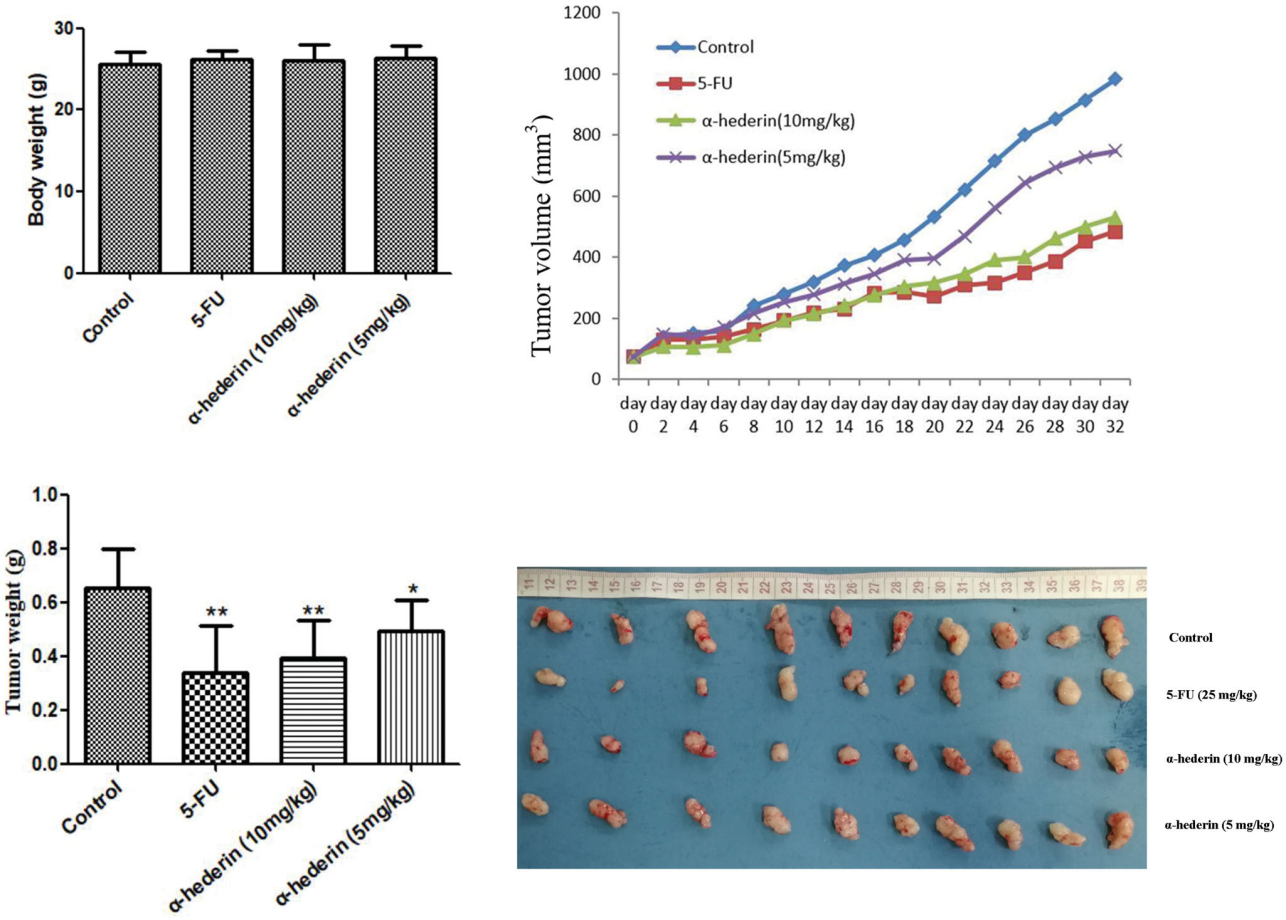
α-Hederin inhibits orthotopic non-small cell lung cancer growth *in vivo*. *p < 0.05, **p < 0.01 as compared to the Normal control group. Mean ± S.E.M. *n* = 10.

The results of this study showed that there was no significant difference in body weight between the groups, and that α-hederin had significant inhibitory effects on tumour volume and tumour weight in xenografted nude mice compared to that in the model group (*p* < 0.05). The average tumour weights of 10 (0.39 g) and 5 mg/kg (0.49 g) α-hederin groups were significantly lower than that of the control (0.65 g), and the inhibitory rates of 10 and 5 mg/kg α-hederin were 40% and 24% (Figure 7).

### Effect of α-hederin on glycolysis-related proteins in transplanted NSCLC A549 tumours in nude mice

Immunohistochemical results showed that compared to that of the model group, the GLUT1 content of NSCLC A549 tumours in the 10 mg/kg α-hederin group was significantly lower (*p* < 0.01) (Figure 8). In addition, GLUT1 expression in the 5 mg/kg α-hederin group was lower, but the difference was not statistically significant. HK2, PKM2, LDHA, MCT4, c-Myc, and HIF-1α expression levels were all significantly lower in the 5 and 10 mg/kg α-hederin groups (*p* < 0.05) (Figure 8).

Figure 8.α-Hederin suppresses expression of glycolysis-related proteins *in vivo*. IHC of GLUT1, HK2, PKM2, LDHA, MCT4, HIF-1α, c-Myc and SIRT6 in tumour tissues (200×). The results showed a remarkable decrease in expression of GLUT1, HK2, PKM2, LDHA, MCT4, HIF-1α, c-Myc and SIRT6 in tumours treatment with 5 and 10 mg/kg α-hederin compared to controls. *p < 0.05, **p < 0.01 as compared to the Normal control group. Mean ± S.E.M. *n* = 6.

**Figure:**
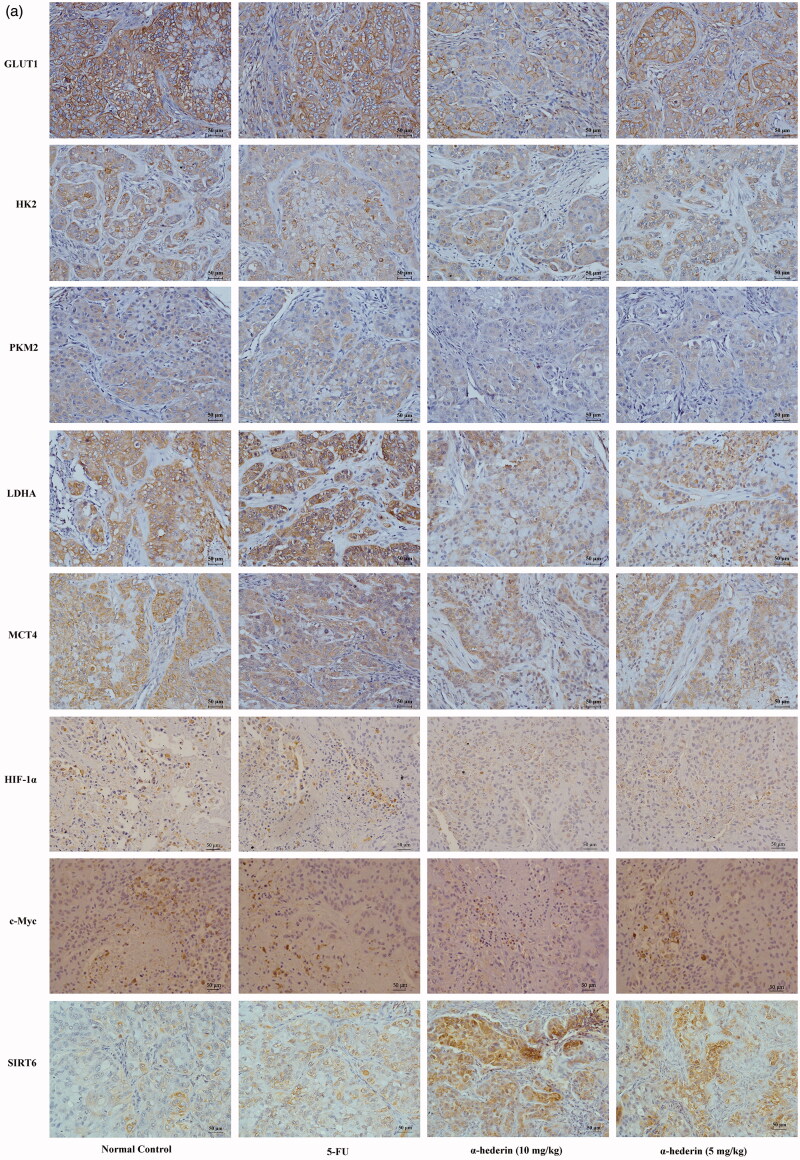

**Figure:**
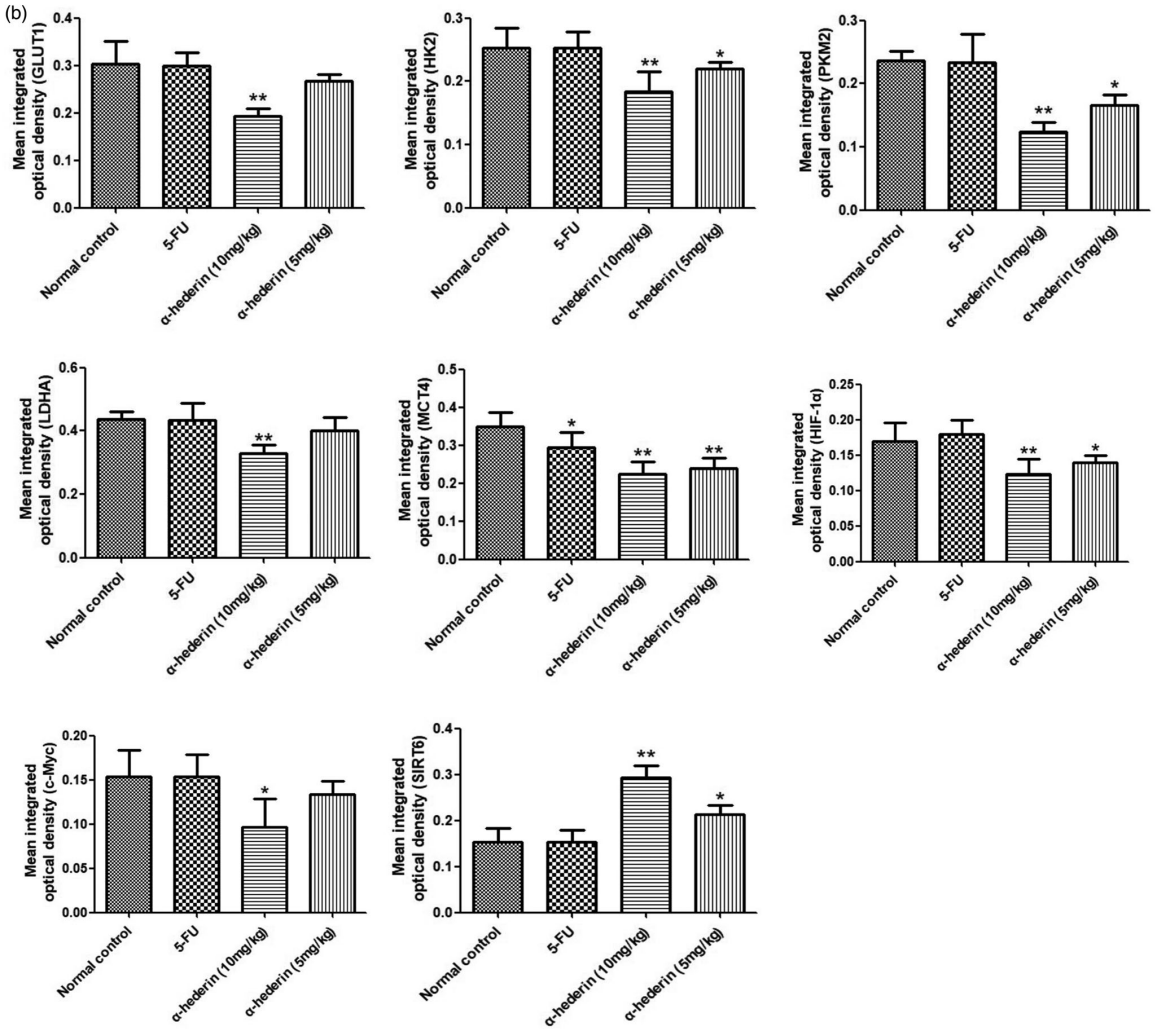

## Discussion

This study assessed the inhibitory effect of α-hederin on tumour cells and measured the effects of glucose, lactate, and intracellular ATP in cell culture medium on glycolysis in A549 cells. Expression of GLUT1, HK2, PKM2, LDHA, MCT4, and glycolytic regulators c-Myc, HIF-1α, p53, Akt, and SIRT6 was detected using western blot assays to determine the inhibitory mechanism of α-hederin on NSCLC A549 cells. We established an NSCLC A549 allograft transplantation tumour model to investigate the effect of α-hederin on xenograft tumours in mice by evaluating tumour volume and weight. In addition, immunohistochemistry was used to detect the expression of glycolysis-related proteins. The results showed that α-hederin inhibited NSCLC A549 *in vivo* and *in vitro* and significantly reduced glucose uptake, reduced lactate generation, and reduced ATP levels in the NSCLC A549 cells. The inhibitory mechanism of α-hederin is mediated by SIRT6, and α-hederin regulates c-Myc and HIF-1α, which reduce the key glycolytic enzymes GLUT1, HK2, PKM2, LDHA, and MCT4.

In the 1920s, Otto Warburg discovered that glycolysis is the primary source of energy for cancer cells, even when oxygen levels are sufficient (Koppenol et al. 2011). Oxidative phosphorylation is the major mechanism by which normal cells produce energy; however, during cell growth and proliferation, tumour cells need a large energy supply in a short time span. Oxidative phosphorylation cannot provide energy for tumours efficiently, whereas the glycolysis pathway can do so quickly. Tumour cell growth depends on a constant supply of energy, and glucose is the primary source of energy for tumour cells. Therefore, cancer cells generate energy primarily via glycolysis, and lactate produced by glycolysis provides an acidic environment for tumour growth (Annibaldi and Widmann 2010). The Warburg effect is manifested primarily by glucose consumption, lactate production, and ATP levels in cancer cells. Therefore, by measuring these three parameters, we can determine whether a drug has an impact on glycolysis. The results of this study demonstrated that α-hederin significantly reduced glucose uptake, lactate production, and ATP levels in NSCLC A549 cells, indicating that α-hederin treatment significantly inhibited glycolysis in A549 cells.

GLUTs are involved in the entry of glucose into cells. GLU1 plays an important role in GLUTs (Kapoor 2013). HK is the initial enzyme of glycolysis and limits the rate of glycolysis. Its subtypes usually have a lower expression level in normal cells but are present in a wide variety of tumour cells at a high expression level. Targeting HK2 can effectively inhibit the growth of tumour cells (Wolf et al. 2011). PKM2 is a key enzyme in glycolysis. It is overexpressed in many malignant tumours and can promote glycolysis in tumour cells (Li et al. 2018). MCT plays an important role in the transport of lactate, pyruvate, and other short-chain monocarbons through membranes (Pérez-Escuredo et al. 2016). LDH is another important regulator of pyruvate and is found in various types of cancer, where its expression and activity are high. LDHA is often involved in aerobic glycolysis in cancer cells (Le et al. 2010). During aerobic glycolysis, initial GLUTs transfer glucose into the cytoplasm. Then, HK catalyses glucose into glucose 6-phosphate and PKM2 catalyses the transfer of glucose 6-phosphate into pyruvate. LDH catalyses pyruvate into lactate, which is finally transported out of the cell by MCTs. The results of this study indicate that α-hederin significantly reduces the expression of the glycolytic proteins GLUT1, HK2, PKM2, LDHA, and MCT4 of NSCLC A549 cells both *in vitro* and *in vivo*.

In cancer cells, GLUT1, HK2, PKM2, LDHA, MCT4, and other glycolysis-related proteins directly affect the glycolytic process. However, these key enzymes are regulated by relevant regulatory factors. Akt plays an important role in cell energy metabolism and may enhance aerobic glycolysis in tumour cells (Polivka and Janku 2014). *p53* is a tumour suppressor gene. The p53 protein reprograms energy metabolism to negatively regulate cell glycolysis by promoting mitochondrial oxidative phosphorylation and inhibiting glycolysis (Matoba et al. 2006; Liang et al. 2013). p53 also regulates GLUTs and glycolysis-related catalytic enzymes to inhibit glycolysis in cancer cells. Expression of GLUT1 and GLUT4 can be directly inhibited and indirectly adjusted by GLUT3 (Schwartzenberg-Bar-Yoseph et al. 2004). p53 also regulates the PI3K/Akt/mTOR pathway, which regulates glucose metabolism (Budanov and Karin 2008). HIF-1α is a subunit of heterodimeric transcription factors that adapt the response of tumour cells to environmental changes in a hypoxic environment. HIF-1α can regulate glucose uptake, glycolytic enzymes, the expression of single carboxylic acid transporters such as GLUT1, GLUT3, HK2, fructose phosphate kinase 2 (PFK2), aldolase A (ALDOA), enolization enzyme (ENO), and pyruvate kinase M (PKM), and the expression of LDHA and MCT 4 (Semenza et al. 1994, 1996; Ullah et al. 2006). c-Myc is a member of the *Myc* gene family of oncogenes and is associated with a wide variety of tumour development. c-Myc promotes glucose absorption by upregulating GLUT1 (Osthus et al. 2000; Dang et al. 2008) and enhancing the transcription of glycolytic enzymes HK2, PFK, and LDHA (Shim et al. 1997; He et al. 2015). It also upregulates the expression of MCT and PKM2 (David et al. 2010; Luan et al. 2015; Gan et al. 2016). In this study, we found that α-hederin significantly reduced the expression of the glycolytic regulators HIF-1α and c-Myc in NSCLC A549 cells but had no significant effect on the expression of p53 or Akt.

SIRT6 plays a key regulatory role in gene transcription, metabolism, maintenance of genomic stability, and integrity of telomeres, thus regulating the occurrence and development of diabetes, obesity, heart disease, cancer, and other diseases. SIRT6 inhibited the activity of the transcription factor HIF-1α and glucose oxidation and glycolysis via the citric acid cycle (Shun et al. 2016). SIRT6 was also found to co-inhibit the transcription activity of the central oncogene MYC involved in ribosome biogenesis (Sebastián et al. 2012). We found that α-hederin significantly increased SIRT6 expression, using the SIRT6 inhibitor OSS_128167 for verification. Our results showed that the expression of HIF-1α and c-Myc was lower when the treatment was OSS_128167 combined with α-hederin than when only OSS_128167 was used. Combined with the results of previous experiments, our results demonstrate that α-hederin activates SIRT6 expression and regulates tumour glycolysis.

## Conclusions

**Figure 9.**
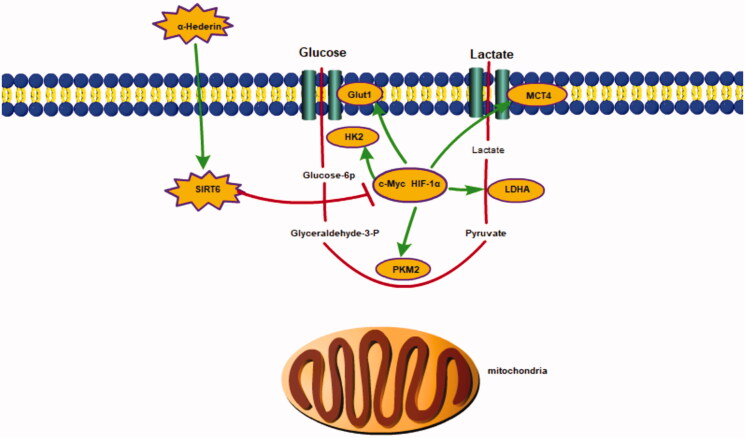
α-Hederin inhibits the growth of lung cancer A549 by decreasing SIRT6 dependent glycolysis.

In our study, α-hederin inhibited the growth of NSCLC A549 cells by suppressing glycolysis. The IC_50_ of α-hederin in A549 cells was 13.75 μM and 10 mg/kg α-hederin had the strongest inhibitory effect on tumour growth in xenografted nude mice. The underlying mechanisms with respect to our findings are that α-hederin activates SIRT6 expression, inhibits the expression of glycolytic regulatory factors HIF-1α and c-Myc, and suppresses the expression of glycolytic proteins (Figure 9).
